# Digital symptom monitoring in cancer care: an exploratory evaluation of low uptake and engagement

**DOI:** 10.1007/s00520-026-11297-3

**Published:** 2026-10-02

**Authors:** Floortje Mols, Meike Hendriks, Nadine Bol, Sophia Sleeman, Olga Husson, Mies van Eenbergen, Corina van den Hurk

**Affiliations:** 1 CoRPS - Center of Research On Psychological Disorders and Somatic Diseases, Department of Medical and Clinical Psychology, Tilburg University, PO Box 90153, 5000 LE Tilburg, The Netherlands; 2 Department of Research & Development, Comprehensive Cancer Organisation (IKNL), Utrecht, The Netherlands; 3 Tilburg Center for Cognition and Communication (TiCC), Department of Communication and Cognition, Tilburg University, Tilburg, The Netherlands; 4 Dutch AYA ‘Young & Cancer’ Care Network, Amsterdam, The Netherlands; 5 Department of Medical Oncology, Netherlands Cancer Institute – Antoni van Leeuwenhoek, Amsterdam, The Netherlands; 6 Department of Surgical Oncology, Erasmus MC Cancer Institute, Erasmus University Medical Center, Rotterdam, The Netherlands; 7 Department of Public Health, Erasmus MC Cancer Institute, Erasmus University Medical Centre, Rotterdam, The Netherlands; 8 Catharina Hospital, Eindhoven, The Netherlands; 9 Santeon, Utrecht, The Netherlands

**Keywords:** AYA, Oncology, Digital symptom monitoring, Real-world implementation, Patient engagement, Systemic treatment, Technology uptake/adoption

## Abstract

**Aim:**

To explore the real-world uptake and engagement with OpenSYMPRO, a digital symptom-monitoring tool for patients with cancer undergoing systemic therapy, and potential barriers to its use.

**Methods:**

The original study aimed to compare adolescents and young adults (AYAs) using openly accessible OpenSYMPRO with patients aged > 40 years using hospital-based regular SYMPRO. OpenSYMPRO was also accessible to older patients. Due to low recruitment, the planned comparative evaluation was abandoned. Engagement was summarized descriptively. All 20 enrolled participants were invited to provide feedback, and all available interviews and questionnaires were analyzed thematically, irrespective of age.

**Results:**

Of 20 enrolled participants, 18 registered through OpenSYMPRO and 2 through regular SYMPRO. Seven completed > 2 symptom questionnaires, 9 completed 1–2, and 4 completed none. Nine participants provided qualitative feedback through interviews (*n* = 4) or questionnaires (*n* = 5), including 7 AYAs and 2 participants outside the AYA age range. Potential barriers included concurrent studies, perceived overlap between studies, forgetting the tool, limited time or cognitive capacity, and limited perceived added value. Some participants found symptom tracking useful. Respondents reported no substantial problems with technical operation, although design-related barriers could not be excluded.

**Discussion:**

Uptake and engagement appeared to be influenced primarily by organizational, communication, and personal factors rather than technical operation. Stronger integration into clinical workflows, person-tailored communication of purpose and added value, active healthcare professional involvement, and attention to potential design barriers warrant further evaluation. Given the small and heterogeneous sample, and limited feedback from non-users and non-participants, findings are hypothesis-generating.

## Introduction

Cancer treatments, including systemic therapy, are often associated with burdensome symptoms that can markedly impair patients’ health-related quality of life (HRQoL) [1]. For adolescents and young adults with cancer (AYAs), who are in a critical phase of personal, social, and professional development, effective symptom management is particularly important as poorly controlled symptoms may hinder education, work, social relationships, and gaining independence [2]. In daily care, symptoms are mainly discussed during regular consultations. However, studies show that healthcare providers (HCPs) miss almost half of patients’ symptoms, leading to suboptimal symptom management [3–5].

Patient-reported outcomes (PROs), defined as direct reports of symptoms from patients without interpretation by others, have therefore gained interest as a complementary source of clinical information [6]. PROs capture a broader range of symptoms than clinician assessments and support shared decision-making and guideline development [7].

Digital health technologies, such as mobile applications, enable real-time PRO monitoring and have demonstrated effectiveness in oncology settings [7, 8]. These tools empower patients, improve communication with HCPs, and support personalized care [9, 10]. Continuous monitoring helps identify symptom patterns and facilitates timely clinical action, such as adjusting medication, modifying treatment, or referring to supportive care [9, 10]. One of the key advantages is the early detection of emerging symptoms, mostly through alerting algorithms. It allows for timely medical intervention before symptoms escalate in severity [9, 10]. In turn, it enhances symptom management, reduces the risk of treatment-related complications, and promotes better treatment adherence, as patients are less likely to discontinue therapy due to intolerable symptoms [7]. Ultimately, digital symptom monitoring has been associated with better symptom control and improved survival outcomes [7, 11–13].

Systematic symptom monitoring through digital health tools has been linked to improved HRQoL [7]. By increasing patients’ sense of control over their health, such tools can reduce uncertainty and anxiety about symptom progression, supporting better psychological well-being [14]. These tools provide accessible, real-time information and generate digital symptom reports to patients displaying the course of symptoms over time, which can guide clinical consultations [7]. Such reports help focus discussions on specific symptom trends, enabling data-driven decisions and more patient-tailored care. This systematic monitoring of symptoms, and the resulting sense of control and involvement in their own care, may contribute to improved PROs.

Another advantage of digital symptom monitoring is that, when alerting algorithms are properly configured, it does decrease unscheduled patient contact. Instead, structured monitoring streamlines communication between patients and HCPs [15–17]. By providing clear guidance on when symptoms require medical attention, these tools can reduce avoidable emergency visits and hospital admissions [18].

To support AYAs during systemic therapy, we developed OpenSYMPRO, a web application for structured symptom tracking based on the existing SYMPRO app, which previously required HCP enrollment [19]. Each year, about 4200 AYAs (18–39 years) are diagnosed with cancer in the Netherlands and treated across multiple hospitals. To ensure broad access, OpenSYMPRO allowed patients to independently create an account without HCP involvement, with a primary focus on reaching AYAs. This study examined the feasibility and acceptability of this open, patient-initiated approach to digital symptom monitoring.

The original aim was to evaluate symptoms, HRQoL, and user experiences among AYAs using OpenSYMPRO and to compare these with those of patients older than 40 years using regular SYMPRO through a participating hospital. OpenSYMPRO was also accessible outside the AYA age range. Recruitment was substantially lower than anticipated, precluding the planned comparisons. The present study therefore reports an exploratory implementation evaluation of the full enrolled sample, including participants outside the AYA age range. It describes uptake and engagement and uses all available qualitative feedback to explore potential barriers and facilitators. The findings are intended to inform future implementation research and are not interpreted as specific to AYAs.

## Methods

### Development and functionality of the open platform app

The SYMPRO app, a digital self-monitoring tool for cancer patients receiving systemic therapy, was developed in 2019 and evaluated in both an RCT and a prospective cohort study [17, 20, 21]. This project aimed to implement the tool in routine practice by making it openly accessible to all patients, regardless of HCP involvement. The primary focus was on AYAs, for whom open access was considered particularly relevant because, in the Netherlands, their treatment is distributed across multiple subspecialities and hospitals and access to AYA-specific supportive care may therefore vary. OpenSYMPRO therefore enabled patients to register independently without requiring initiation by an HCP.

Unlike regular SYMPRO, patients could self-register for the OpenSYMPRO app without HCP involvement. From the first login, they provided informed consent for research use of their data, which were securely stored within the Patient Reported Outcomes Following Initial treatment and Long term Evaluation of Survivorship (PROFILES) registry [22], also hosting the SYMPRO app. As OpenSYMPRO was not linked to hospitals, symptom alerts were not forwarded to HCPs. Instead, participants were advised to contact their HCP if alerts occurred and to discuss results during consultations, following the ‘reactive’ model previously applied in SYMPRO [23].

Another difference from SYMPRO was that OpenSYMPRO was tailored to systemic treatments commonly used in AYAs through linkage with the Dutch informational website on cancer symptoms for patients (www.bijwerkingenbijkanker.nl) [21]. After selecting their treatment, participants received a treatment-specific symptom list with optional free-text fields for additional symptoms and could access related information about the symptoms directly in the app. Cancer type–specific symptoms (like cough for lung cancer) were not included, given the wide variety of tumors occurring in this age group.

The core interface and functionality of the existing SYMPRO platform were retained and were not specifically redesigned or co-designed for different developmental stages within the AYA age range. Tutorials and digital materials were developed to support app use in collaboration with the Space4AYA patient organization, and a PROFILES helpdesk assistant was available during office hours. The app was accessible on all mobile devices and free of charge during the project.

At first login, users completed a short intake on diagnosis and treatment (chemotherapy, immunotherapy, hormonal, or targeted therapy). They then received weekly push notifications or emails to complete a brief symptom questionnaire (± 5 min), at least weekly during the first year after diagnosis, with a maximum of 1 entry per day. Questions were based on the validated Dutch Patient-Reported Outcomes Common Terminology Criteria for Adverse Events (PRO-CTCAE) [24–26], covering symptom severity, frequency, and interference. Patients rated symptom severity in the past week, could add additional symptoms, or indicate experiencing no symptoms. Computerized adaptive testing skipped follow-up questions if no symptoms were reported or were not applicable.

Alerts to contact an HCP were triggered by predefined conditions based on healthcare risk or specific symptom patterns, similar to SYMPRO [17, 21]. Information materials emphasized that hospital-specific instructions (e.g., for fever) always prevailed over app notifications.

Questionnaire responses generated reports on symptom severity, progression, and alerts. Answers on 5-point Likert scales were converted to PRO-CTC sum scores [27] and displayed in 3 tabs: (1) alert-triggering symptoms, (2) overall scores per symptom, and (3) item-level details. Patients were encouraged to share these reports with their HCPs during consultations.

### Patient population

The original study defined AYAs as patients aged 18 to 40 years at diagnosis and planned to compare AYAs using OpenSYMPRO with patients older than 40 years using regular SYMPRO through a participating hospital. OpenSYMPRO did not restrict registration by age, and participants older than 40 years using OpenSYMPRO were originally intended to form an additional comparison group. The present exploratory evaluation includes all enrolled participants, irrespective of age. We report the sample by age at diagnosis, distinguishing diagnoses before age 18, at ages 18 to 40, and after age 40. Age at diagnosis should be distinguished from age at study participation. Eligible participants had a cancer diagnosis at any stage, were starting systemic treatment (chemotherapy, immunotherapy, hormonal therapy, or targeted therapy), were Dutch-speaking, and had internet access. No exclusion criteria applied.

Participants could enter the study through two routes. OpenSYMPRO was openly accessible after finding the tool through leaflets distributed across hospitals, posters, social media, news items, or the PROFILES website. Regular SYMPRO was available to patients aged > 40 years enrolled through a participating hospital, where HCPs registered patients and provided them with login details. For both groups, the reactive alert model was applied [23]. For patients enrolled through regular SYMPRO in the participating hospital, HCPs could access patients’ reports through the PROFILES registry.

Patients interested in OpenSYMPRO could register via email and immediately access the app after providing digital informed consent. App use was linked to participation in this study, which was reviewed by an official medical ethics committee (METC Brabant, the Netherlands) and declared exempt from the Medical Research Involving Human Subjects Act (non-WMO declaration, NW2021-76). This study was performed in compliance with the Declaration of Helsinki. Data collection complied with GDPR regulations. Participants were informed about data use before consenting and could withdraw from the study at any time without consequences.

### Assessment methods

In addition to weekly symptom questionnaires, participants completed 4 study questionnaires. At baseline, they filled out a 35-min intake assessing sociodemographic and clinical characteristics (e.g., age, cancer type, comorbidities, prior treatments, treating institute, and recruitment source), as well as HRQoL (European Organization for Research and Treatment of Cancer Quality of Life cancer survivorship core questionnaire (EORTC QLQ-SURV100) [28]). After 3 months, a 15-min usability and experience questionnaire evaluated alert handling, perceived usefulness, improvement suggestions, and willingness to recommend the app. Follow-up questionnaires on symptoms and HRQoL (EORTC QLQ-SURV100) were administered at 6 and 12 months (≈20 min each).

To better understand user experiences and barriers to OpenSYMPRO use, we conducted interviews with frequent, infrequent, and non-users (Appendix). Participants could take part by phone; those declining an interview were offered a brief questionnaire instead. Including all user types allowed exploration of both how the app was used and why some patients chose not to use it. All 20 enrolled participants were invited to provide qualitative feedback, irrespective of age or level of app use. All available feedback was included; participants were not selected for the qualitative analysis based on AYA status.

### Shift in study focus

Recruitment was substantially lower than anticipated despite multiple efforts to enhance engagement. Consequently, the prespecified comparative analysis plan was abandoned, as the planned comparisons between AYAs and participants aged > 40 years with respect to symptoms, HRQoL, and app experiences were considered neither statistically meaningful nor appropriate given the small sample. The study objective was therefore revised to explore potential reasons for low uptake and engagement in the full enrolled sample, which included participants both within and outside the AYA age range. To this end, qualitative data were subsequently collected through interviews and questionnaires to identify potential barriers and facilitators to uptake and use. No inferential comparisons between age groups were made.

### Analysis

Quantitative outcomes were summarized descriptively: counts/percentages for categorical variables and medians (IQR) for continuous variables. All available qualitative feedback from frequent users, infrequent users and participants who did not complete symptom questionnaires was analyzed together by MH and FM to identify potential barriers, facilitators and implementation considerations. The analysis included respondents both within and outside the AYA age range. Themes were interpreted at the level of this combined sample; no separate thematic analysis or comparison by age group was undertaken.

## Results

### Recruitment and deviations from the original plan

After 18 months of OpenSYMPRO availability and 6 months of regular SYMPRO use in one hospital, 20 participants had enrolled by logging in at least once. This was the full study sample, including participants both within and outside the AYA age range. Eighteen enrolled through OpenSYMPRO and 2 through regular SYMPRO in the participating hospital; neither of the latter completed any questionnaires. Despite careful development and communication about the app through several channels, uptake and engagement were low: 7 patients were frequent users (> 2 questionnaires), 9 used the app only once or twice, and 4 never filled out questionnaires. Age at diagnosis was available for 19 out of 20 participants. Of the full sample, 15 were diagnosed at ages 18 to 40 years (range 21 to 40), 3 after age 40 (range 58 to 76), and 1 before age 18. The participant diagnosed before age 18 may have enrolled years later, possibly following cancer recurrence or a new primary cancer. Since the reason for treatment at enrollment was not available, this explanation could not be verified. Age at study participation was not available, limiting interpretation of participants’ life stage during the evaluation. Participants could potentially have been considerably older at the time of study participation. Among the 16 participants who actively completed questionnaires, 11 (69%) received at least 1 alert. In total, 67 alerts were generated, with an average of 4.2 alerts per active participant, and the highest number of alerts for a single participant was 16.

All 20 enrolled participants were invited to provide feedback through a telephone interview or an online questionnaire, irrespective of age. Nine responded: 4 participated in an interview (age not registered) and 5 completed a questionnaire (4 AYAs and 1 over 40 at participation). Four were frequent users, 2 were infrequent users and 3 had not completed any symptom questionnaires. All 9 respondents’ accounts were included in the thematic analysis. The themes therefore reflect this combined sample and are not interpreted as specific to AYAs.

### Factors potentially influencing engagement with OpenSYMPRO

Thematic analysis of the 4 interviews and 5 questionnaires from all 9 respondents identified 5 recurring factors that may have influenced app use or continued engagement. These themes reflect a small, mixed-age sample and should not be interpreted as specific to AYAs or representative of patients with cancer more broadly. The identified themes often overlapped and included five potential barriers: (1) participation in multiple concurrent studies, (2) perceived study overlap, (3) forgetting or lack of recall, (4) limited time or cognitive capacity, and (5) perceived lack of added value. In addition, participants generally reported few problems with the technical operation of the platform. Each theme is described below and illustrated with translated quotations from interviews and questionnaires.

### Multiple concurrent studies

Several participants reported feeling overloaded by invitations to participate in multiple studies and digital tools during their cancer trajectory. The abundance of invitations, questionnaires and (digital) tools caused confusion about each initiative’s purpose and relevance, making OpenSYMPRO seem like just another study rather than part of their care. This cognitive burden appeared to contribute to selective participation or discontinuation, particularly when the tool was not perceived as directly beneficial.

I get called by so many different people. I honestly do not even remember who is from which study anymore. (Participant 1, female)

### Perceived overlap across studies

Some participants felt that OpenSYMPRO questions closely resembled those from other studies or digital tools, creating a sense of repetition and frustration. This overlap reduced motivation to engage with yet another platform perceived as offering little added value. As one participant noted: “These questionnaires all feel very similar. I think I have answered the same things multiple times.” (Participant 6, female).

### Forgetting or a lack of recall

Several participants had completely forgotten about the OpenSYMPRO app or no longer remembered its purpose. Participants’ accounts suggest that digital tools may easily fade from attention when they are not clearly connected to routine care or when reminders are overlooked. Many did not associate the app with their care team or clear benefits to their treatment or well-being, contributing to low engagement.

I honestly have no idea what it [OpenSYMPRO] was about or why you are calling me. (Participant 2, female)

### Limited time or cognitive capacity

Many participants described their cancer treatment period as intense and emotionally overwhelming. Although several initially consented to participate in OpenSYMPRO, they were unable to continue due to the demanding nature of treatment. Even minor tasks, such as completing a questionnaire, were often perceived as burdensome. Remembering use the app or finding the energy to engage was challenging amid medical appointments, physical symptoms, and emotional stress, leaving little capacity for additional activities.

A lot was happening at the time, and I just could not really deal with anything extra. (Participant 4, female)

### Perceived lack of added value

Some participants did not perceive the OpenSYMPRO app as useful or necessary during treatment. One noted that chemotherapy-related symptoms were too severe to forget, reducing the need for an additional tool to support symptom recall. Another felt that being reminded of unexperienced potential symptoms was distressing. Several respondents also believed they could communicate their symptoms effectively to HCPs without the app. Overall, these experiences suggest that OpenSYMPRO offered limited added value for symptom management or interaction with the care team.

If I do not experience a particular side effect, I do not want to be made aware that it could occur. (Participant 5, female)

### Technical operation and usability

Most respondents indicated that the technical operation of the OpenSYMPRO web application was manageable and acceptable, and no substantial technical problems were reported. However, one participant indicated that a downloadable mobile app would have been more convenient than a web application. This relates more to accessibility and design than to the technical functioning of the platform.

No, the tech part is fine. It works well enough. (Participant 7, male)

### Positive experiences and facilitators of use

Although respondents provided considerably more information about barriers than facilitators, some positive experiences were reported. Among respondents providing feedback, the platform was generally considered easy to use, and its technical operation was not identified as a reason for discontinuation. For some participants, tracking symptoms also provided useful insight into their symptom burden. Given the limited number of respondents, these observations should be considered descriptive.

It helps to make clear what symptoms you are experiencing. (Participant 8, female)

## Discussion

### Recommendations for implementation and uptake

This study was originally designed to evaluate OpenSYMPRO among AYAs and compare their experiences and outcomes with those of patients older than 40 years using regular SYMPRO. Low recruitment precluded these comparisons. The present evaluation therefore describes uptake and engagement in the full enrolled sample and draws on qualitative feedback from 9 respondents, including 7 AYAs and 2 participants outside the AYA age range. Their accounts suggested potential barriers involving competing research demands, perceived overlap, forgetting the tool, limited capacity during treatment, and limited perceived added value. Respondents reported few problems with the platform’s technical operation, although design barriers cannot be excluded. These exploratory findings relate to the combined sample. They neither establish barriers specific to AYAs nor demonstrate that the same barriers occur across age groups.

One factor that may be particularly relevant for implementation is the extent to which symptom monitoring is integrated into clinical care. Most participants in the present study entered through OpenSYMPRO and therefore enrolled independently, with limited direct HCP involvement; only 2 participants were enrolled through regular SYMPRO in the participating hospital. Participants’ accounts suggest that this limited connection with clinical care may have contributed to low engagement. This interpretation is consistent with previous literature emphasizing the importance of clinical integration of PRO monitoring [18], but cannot be established causally from the present exploratory data.

Building on these exploratory findings, several strategies warrant further evaluation to support initial uptake and sustained engagement. These include minimizing questionnaire burden, communicating the purpose and added value of symptom monitoring on an individual level, distinguishing from or integrating the tool in concurrent studies or digital initiatives, and strengthening its connection with clinical care. We discuss these strategies below.

### Streamline coordination, reduce overlap, and clarify unique contributions

Participants reported confusion and burden when multiple digital initiatives were introduced simultaneously without clear distinction. In OpenSYMPRO, additional study questionnaires increased workload and reduced engagement. To prevent duplication, research teams and clinicians should coordinate introductions and requests, ensuring patients receive coherent and minimal tasks. Local alignment, such as hospital registries of ongoing PRO or digital studies, can facilitate collaboration and data sharing, and reduce redundancy. Each tool should have a clearly defined purpose and benefit, distinct from other initiatives. Overlap in content should be minimized or transparently explained. Where possible, harmonizing data collection across studies—ideally within a single integrated app—can further reduce burden, enhance relevance, and improve usability.

### Integrate digital tools more clearly into routine care

A recurring theme in the interviews was the perceived detachment of OpenSYMPRO from participants’ regular care. Many did not recognize how the app related to their treatment trajectory or healthcare team. The available patient information may not have communicated the purpose and added value of OpenSYMPRO sufficiently clearly, although we cannot determine whether or how participants engaged with these materials. Although the benefits of digital symptom monitoring are well established in trials and observational studies, successful implementation in routine care is likely to benefit from integration into clinical workflows. Initiatives such as iPEHOC, used by thousands of patients, demonstrate that engagement starts with HCPs who understand and support the added value [29]. Active involvement of HCPs may help strengthen communication and support the triage and management of treatment-related side effects.

### Sustain engagement through active follow-up

Many participants lost track of the app despite weekly email reminders. Embedding it in an app store could improve visibility and enable push notifications. Privacy regulations also limited reminder options for those who had shown interest but had not yet consented. To sustain engagement, passive email reminders should be complemented by active, personalized strategies linked to care processes—such as referencing the tool during consultations, aligning reminders with treatment milestones and tying its use to concrete treatment decisions [30]. Successful implementation depends on adoption by HCPs, a known challenge in PRO use [18]. Ideally, reminders originate from familiar hospital systems. In the Netherlands, these platforms are already extensively used by patients to check appointments and results of medical examinations, and access online patient information.

### Improve timing and flexibility of implementation

The treatment phase is often marked by emotional distress, fatigue, and competing demands, making even low-threshold interventions feel burdensome. Brief, non-intrusive check-ins from the care team can help maintain awareness of digital tools. Flexible participation models, allowing patients to opt in at different stages or pause use during intensive treatment, may better align with patients’ lived experiences.

### Technical considerations

Participants who provided feedback generally did not identify the technical operation of the platform as a major barrier. However, this does not exclude potential design-related barriers among individuals who did not register or who disengaged early. For example, OpenSYMPRO was offered as a web application rather than a downloadable mobile app, which may have affected its visibility, accessibility, or perceived attractiveness. More broadly, aspects such as look and feel, navigation, and the number of steps required to access or use the tool were not systematically evaluated in the present study.

Patient-facing usability should be distinguished from integration into healthcare systems. OpenSYMPRO operates within the PROFILES registry but is not currently directly integrated into hospital electronic health records. The technical feasibility and complexity of such integration were not assessed in this study. Integration with hospital systems and clinical workflows therefore remains an important consideration for future implementation.

### Age and life stage considerations for future implementation

The original focus on AYAs raises questions about age and life stage that the present mixed age evaluation could not answer. The following considerations are directions for future research, rather than findings specific to AYAs from this study. Implementation strategies may need to account for heterogeneity within populations. Individuals in late adolescence or early adulthood may differ substantially from those approaching 40 years in developmental stage, family responsibilities, employment, healthcare needs, and expectations of digital technology. Our sample was too small to examine whether engagement or perceived barriers differed across these life stages. Moreover, although OpenSYMPRO was adapted to facilitate independent access and provide treatment-specific symptom content, its core interface was not specifically co-designed for AYAs or for different developmental subgroups within the AYA population. We therefore cannot exclude that design-related factors, including the platform’s look and feel, navigation, or perceived attractiveness, affected uptake or engagement. Future implementation studies could examine whether needs and preferences differ by age and life stage and involve more diverse AYAs directly in co-design in an earlier stage of development.

### Limitations

Several limitations should be considered. First, the sample was very small, and qualitative feedback was obtained from only 9 participants. Data saturation cannot be assumed, and the identified themes may not represent the full range or relative importance of barriers and facilitators in the population targeted by this intervention. Second, the sample was clinically and developmentally heterogeneous, while its size precluded meaningful analyses according to age or life stage, cancer diagnosis, treatment, or phase of care. Third, the sample included participants outside the AYA age range, and the qualitative analysis combined feedback from 7 AYAs and 2 participants outside that range. These findings describe the combined sample and cannot establish which barriers, if any, are specific to AYAs. Furthermore, age at diagnosis does not necessarily reflect age or developmental stage at study participation. Fourth, and importantly, we could only obtain feedback from individuals who registered or otherwise entered the study. Patients who encountered the intervention but chose not to register could not be approached, meaning that the present data provides substantially more insight into engagement and discontinuation than into initial non-uptake. Future implementation studies should therefore prospectively include methods to investigate initial non-uptake, for example by obtaining brief anonymous feedback from eligible patients who decline registration or by systematically recording reasons for declining when the tool is introduced in clinical practice. Fifth, feedback focused predominantly on barriers, providing limited information on factors facilitating use. Finally, the absence of substantial reported problems with the technical operation of the platform among respondents does not exclude design-related barriers among individuals who did not register or who disengaged early. Such barriers may include visibility, accessibility, navigation, look and feel, or the number of steps required to engage with the platform. These patient-facing design considerations should also be distinguished from the separate challenge of integrating the platform into hospital electronic healthcare record systems and clinical workflows.

## Conclusion

This exploratory evaluation describes low uptake and engagement in a digital symptom monitoring initiative originally focused on AYAs but accessed by a mixed age group. Qualitative feedback from 9 respondents suggested potential barriers related to competing research demands, perceived overlap, forgetting the tool, limited capacity during treatment, and limited perceived added value. These findings describe the combined sample and do not establish barriers specific to AYAs. The small sample and absence of feedback from people who never registered also limit conclusions about initial uptake. Future implementation studies should prospectively evaluate whether closer integration into clinical care, active HCP involvement, clearer communication of purpose and added value, and reduced participant burden support uptake and sustained engagement. Whether additional adaptations are needed for AYAs or other age and life stage groups requires further study.

## Data Availability

Not applicable.

## Appendix. The interview guide for three distinct user groups

1. Non-users (gave consent, but never used the app)
  - What made you decide not to use the OpenSYMPRO app after learning about it?
  - Was there anything that discouraged you from starting (e.g., complexity, privacy concerns, lack of time)?
  - Would you consider using such an app in the future? Why or why not?
  - Is there anything else you would like to share about the app?
2. Low-frequency users (used the app once or twice only)
  - What motivated you to initially use the OpenSYMPRO app?
  - Why did you stop using the app after one or two questionnaires?
  - Was there anything in the app that you found unclear, time-consuming, or unhelpful?
  - What improvements or support might help you re-engage with the app?
  - Is there anything else you would like to share about the app?
3. Frequent users (used the app regularly)
  - What was your first impression of the app?
  - Has the app helped you better understand your symptoms or communicate them to your care team? If so, how?
  - How easy do you find the use of the app? (Very easy/Easy/Neutral/Difficult/Very difficult)
  - Which features do you use most often (e.g., symptom tracking, notifications, reporting)?
  - Are there features you feel are missing or could be improved?
  - Have you shared the app’s reports or data with your healthcare provider? If not, what held you back?
  - Would you recommend the app to other AYA cancer patients? Why or why not?
  - Is there anything else you would like to share about the app?

## Abbreviations

*AYA*: Adolescent and young adult
*HCPs*: Healthcare providers
*HRQoL*: Health-related quality of life
*PROFILES*: Patient Reported Outcomes Following Initial treatment and Long term Evaluation of Survivorship
*PROs*: Patient-Reported Outcomes
*PRO-CTCAE*: Patient-Reported Outcomes Common Terminology Criteria for Adverse Events
*EORTC QLQ-SURV100*: European Organisation for Research and Treatment of Cancer Quality of Life cancer survivorship core questionnaire
*RCTs*: Randomized controlled trials
